# Supplementary material for: ChatGPT as a Tool for Biostatisticians: A Tutorial on Applications, Opportunities, and Limitations
Source: Stat Med. 2025 Oct 23;44(23-24):e70263. doi: 10.1002/sim.70263 (PMC12548020; doi:10.1002/sim.70263)
Supplement: Supplementary file 1 — Data S1. Supporting Information. [file SIM-44-0-s001.zip › Supplementary Materials Additional.zip!/5.1_Variability_in_Chats.pdf]

OpenAI

Biostatistics Model Variability

In each use case, the team produced 10 ChatGPT reactions on the same set of prompts.
There was variability within each set of answers which may be of interest from a statistical or analytical perspective.
Several types of insights could be derived. First, one can learn about epistemic variability (Answer Variance).
Variations may suggest that the questions are ambiguous, ChatGPTs knowledge is broad but not definitive on the topic,
or the subject matter allows for open interpretation (methodological creative questions). This allows to gauge the epistemic uncertainty or answer stability of the language model.
A second point relates to semantic clustering. This is related to the
point of how many distinct perspectives ChatGPT offers on a single question. One could
explore whether the ten answers form distinct clusters (e.g., conservative vs. speculative, technical vs. conceptual).
Third, the variability elucidates stochastic behavior. Since ChatGPT operates probabilistically (unless temperature = 0), one could analyze:
How much randomness influences the response, how different temperature settings affect answer diversity. This would allow to
explore the relationship between model temperature, prompting, and output variability.
The fourth issue is robustness and bias detection. By comparing the responses, one can identify systematic tendencies or biases in how the model frames answers.
It is of interest, whether certain narratives or perspectives are consistently favored.
The fifth point relates to prompt engineering sensitivity. The material can be used totest how sensitive the model is to small changes in the prompt,which is crucial for prompt engineering. It would be iof interest to determine where the boundary lies between stable and volatile model outputs.
Can you provide me with an examplke for the points mentioned above from biostatistics?

Certainly, Ulrich. Here's a concrete example from **biostatistics** that illustrates each of the five points you mentioned—*epistemic variability*, *semantic clustering*, *stochastic behavior*, *robustness/bias*, and *prompt sensitivity*. We'll base the example on a relatively open-ended yet technically grounded prompt:

Prompt:

> "What are appropriate statistical models for analyzing survival data in clinical trials?"

---

1. Epistemic Variability (Answer Variance)

Generate 10 responses to the prompt above. You might find that some answers mention:

- Cox proportional hazards model
- Accelerated failure time models

- Parametric survival models (Weibull, exponential, log-logistic)
- Competing risks models
- Frailty models
- Machine learning approaches (e.g., random survival forests, DeepSurv)

Insight: The variability reflects the **breadth of valid modeling approaches**, as well as **uncertainty about the best choice** absent further context (e.g., proportionality assumption, censoring mechanism). If ChatGPT sometimes prioritizes Cox models and other times goes directly to parametric or nonparametric techniques, that variance reflects *epistemic uncertainty* in the model's prioritization of knowledge.

---

2. Semantic Clustering

Among the 10 answers, you may observe **clusters** like:

- **Classical statistical models**: Cox, Weibull, Kaplan-Meier
- **Bayesian methods**: Bayesian survival regression, hierarchical modeling
- **ML approaches**: Random forests, neural networks
- **Interpretation-focused**: Emphasis on proportional hazards assumption, censored data issues

Insight: This clustering shows the **distinct "perspectives"** ChatGPT can take—technical, methodological, Bayesian, ML-based, etc. Cluster analysis could be performed (e.g., using embeddings) to **quantify the diversity of interpretive frames**.

---

3. Stochastic Behavior (Randomness Influence)

If you run the same prompt with different temperature settings:

- **Temp = 0.2**: Almost all responses converge on Cox models and mention standard techniques.
- **Temp = 0.8**: You see more diversity—some mention deep learning, novel research approaches, or cite recent Bayesian advancements.

Insight: This gives a **quantitative handle on how randomness modulates answer diversity**. You can measure entropy or topic coverage across temperatures to gauge stochastic effects.

---

4. Robustness and Bias Detection

You might observe that, across all runs, the **Cox proportional hazards model is always mentioned first** or disproportionately emphasized.

Insight: This may reflect **training corpus bias** (e.g., frequent co-occurrence of "survival analysis" with "Cox model"). Similarly, if Bayesian models are rarely mentioned, despite being common in some modern biostatistics contexts, this asymmetry reveals **model preference bias or imbalanced representation**.

5. Prompt Engineering Sensitivity

Try variations like:

- "What are advanced models for survival analysis in oncology?"
- "Compare Cox and AFT models for survival data."
- "What are deep learning approaches to survival modeling?"

Insight: You'll likely see **shifts in answer content and prioritization** depending on wording and specificity. This can help define the **prompt volatility threshold**—the point at which a small rephrasing leads to qualitatively different answers.

You could quantify this using response similarity metrics (e.g., cosine similarity over embeddings).

Summary Table

Analytical Point	Key Biostatistical Manifestation in Example
**Epistemic Variability**	Different legitimate survival models listed in different runs
**Semantic Clustering**	Conceptual groupings: Classical, Bayesian, ML, assumption-focused
**Stochastic Behavior**	Greater model diversity with higher temperature
**Bias/Robustness**	Repeated emphasis on Cox model suggests corpus-derived model bias
**Prompt Sensitivity**	Small changes in wording shift model prioritization and specificity

---

Would you like me to simulate a few example ChatGPT responses to this prompt so you can see the variation directly?
